# The Smoke-Free Law’s Toll on Intensive Psychiatric Care Unit (IPCU) Staff

**DOI:** 10.1192/bjo.2025.10498

**Published:** 2025-06-20

**Authors:** Praveen Kumar, Ananya Santosh, Caio Bezzerraculas, Nikki Thomson

**Affiliations:** 1 City Hospital, Aberdeen, United Kingdom; 2 New Craig’s Psychiatric Hospital, Inverness, United Kingdom

## Abstract

**Aims:** This survey aimed to evaluate the impact of the Smoke-Free Perimeter Law on the staff working in the IPCU at New Craig’s Psychiatric Hospital. It focused on understanding how the law affected staff workload, their experiences with patient care, and the overall working environment in the IPCU.

**Methods:** A survey was conducted involving IPCU staff members, comprising various roles with diverse experiences ranging from 2 to over 30 years in psychiatric care. The survey included questions about changes in workload, patient behaviour, staff stress levels, and challenges faced due to the law. Open-ended questions allowed staff to provide detailed feedback and suggestions for improvement.

Results:

Workload and Staff Experiences:

All nine respondents reported a significant increase in workload, primarily due to the additional responsibilities related to managing smoking breaks for patients.

Staff observed notable changes in patient behaviour, including increased physical and verbal aggression, less tolerance, and more frequent aggressive outbursts.

Many patients who were restricted from off-ward smoking breaks exhibited increased irritability and agitation.

Challenges and Environmental Impact:

Managing patient distress and aggression became more challenging, especially when unable to facilitate timely off-ward smoking breaks.

Designated times for escorted smoking breaks led to inconvenience and heightened patient emotions, often resulting in aggression.

The inability to use the courtyard for smoking negatively impacted the ward environment, leading to increased stress and confrontations.

Staff Opinions and Feedback:

Some staff expressed support for a smoke-free hospital but acknowledged the challenges for detained patients.

Concerns were raised about the fairness of enforcing a smoking ban on involuntary patients.

The previous practice of using the courtyard for smoking was seen as beneficial for calming patients and maintaining a closer staff presence.

Training and support needs were mixed, with some staff requesting more support to manage patient aggression and distress.

**Conclusion:** The survey findings illustrate the significant impact of the Smoke-Free Perimeter Law on staff at the IPCU. The increased workload, heightened stress levels, and challenges in patient management highlight the practical difficulties in implementing this policy in a psychiatric setting. Staff feedback underscores the need for supportive measures and potential adjustments to the law’s implementation, ensuring it accommodates the unique needs of both patients and staff. Balancing the implementation of public health policy with the immediate needs of psychiatric patients and staff remains a complex, yet crucial, endeavour in ensuring effective and compassionate psychiatric care.

